# Facial height in Japanese-Brazilian descendants with normal occlusion

**DOI:** 10.1590/2176-9451.19.5.054-066.oar

**Published:** 2014

**Authors:** Fabiano Paiva Vieira, Arnaldo Pinzan, Guilherme Janson, Thais Maria Freire Fernandes, Renata Carvalho Sathler, Rafael Pinelli Henriques

**Affiliations:** 1 Professor, Federal Institute of Paraná; 2 Associate professor, Department of Pediatric Dentistry, Orthodontics and Collective Health, School of Dentistry - University of São Paulo/Bauru; 3 Full professor, Department of Pediatric Dentistry, Orthodontics and Collective Health, School of Dentistry - University of São Paulo/Bauru; 4 Professor, University of Northern Paraná (UNOPAR); 5 Professor, Hospital for Rehabilitation of Craniofacial Anomalies/USP; 6 Professor, Central-West College Pinelli Henriques

**Keywords:** Orthodontics, Ethnic groups, Vertical dimension

## Abstract

**OBJECTIVE:**

The aim of this study was to determine the standards of facial height in 30 young (14-year-old) Japanese-Brazilian descendants with normal occlusion, and assess whether sexual dimorphism is evident.

**METHODS:**

The cephalometric measurements used followed the analyses by Wylie-Johnson, Siriwat-Jarabak, Gebeck, Merrifield and Horn.

**RESULTS:**

Results showed dimorphism for total anterior facial height (TAFH), lower anterior facial height (LAFH), anterior facial height (AFH), total posterior facial height (TPFH) and upper posterior facial height (UPFH) measurements.

**CONCLUSIONS:**

The standards of facial heights in young Japanese-Brazilian descendants with normal occlusion were observed. Sexual dimorphism was identified in five out of thirteen evaluated variables at this age range.

## Abstract (pt)

**INTRODUÇÃO:**

o objetivo desse estudo foi obter os valores médios de normalidade das alturas faciais anterior e posterior de 30 jovens mestiços nipo-brasileiros, descendentes de xantodermas e leucodermas, com oclusão normal, com idade média de 14 anos, e verificar a presença ou ausência de dimorfismo entre os sexos.

**MÉTODOS:**

elaborou-se um estudo cefalométrico com as mensurações advindas das análises de Wylie e Johnson, Siriwat e Jarabak, Gebeck, Merrifield e Horn.

**RESULTADOS:**

os resultados revelaram a presença de dimorfismo entre os sexos para as variáveis AFAT, AFAI, AFA, AFPT e AFPS.

**CONCLUSÕES:**

um padrão cefalométrico específico, das alturas faciais anterior e posterior, para os jovens mestiços nipo-brasileiros com oclusão normal, descendentes de xantodermas e leucodermas, foi apresentado e um dimorfismo entre os sexos para cinco das treze variáveis avaliadas foi verificado nessa faixa etária média.

## INTRODUCTION

Within the context of contemporary Orthodontics, making accurate diagnosis and prognosis determines whether a clinician can provide patients with the best cost-benefit treatment. Based on principles of effectiveness and efficiency, only one or two treatment alternatives best fit patient's esthetic, functional and psychological needs.^1^ Therefore, clinicians must use all possible resources to achieve this ideal goal on orthodontic practice. 

Cephalometry is a valuable auxiliary diagnostic tool as it allows the relationship among bone structures, dental tissue and soft tissue to be determined by means of lateral radiographs,^6^ thereby facilitating complete assessment of malocclusion in different space dimensions, including anterior-posterior^25^ and vertical.^30^ Analyzing malocclusion this way allows understanding of how and in what direction and manner each element of the stomatognathic system contributes to its conformation. For this reason,the use of cephalometric analysis is rendered necessary in the anterior-posterior and vertical directions, and so it is to analyze the influence of vertical changes in the severity of malocclusion in anterior-posterior direction.^23^

Vertical facial changes influence mandibular position and rotation, either clockwise or counterclockwise, thereby contributing to the development of deep or open bite. Thus, orthodontic treatment should induce desirable changes and minimize inevitable undesirable ones.^22^

To assess vertical facial changes, new cephalometric analyses were developed,^24^ and the present study uses measurements employed by Wylie and Johnson,^30^ Siriwat and Jarabak,^24^ Gebeck,^8^ Merrifield^17^ as well as Horn,^9^ all of which assess anterior and posterior facial height, facial ratios and facial height index, measurements which were used in previous studies.^5^^,^ 26 In these studies, cephalometric standards from different racial and ethnic groups and with miscegenation were determined and compared, showing the need for individualization, which has also been reported in worldwide literature.^5^^,^ 7 ^,^ 13 ^,^ 18 ^,^ 26

The studies found in the literature were devoted to certain groups, such as Caucasians or Mongoloids, but not to the result of their miscegenation. For this reason, explaining the need for individualization and understanding of cephalometric characteristics of different miscegenation patterns is important.

To this end, the following were assessed: Specific cephalometric patterns of anterior and posterior facial heights; facial ratios and facial height index^9^ for young Japanese-Brazilian descendants with normal occlusion using variables in the vertical direction of the face. The presence of sexual dimorphism was also assessed.

## MATERIAL AND METHODS

A total of 30 lateral cephalometric radiographs of young Japanese-Brazilian descendants (15 males and 15 females with an average age of 14 years) with normal occlusion, selected from elementary and high schools located in the city of Bauru, were assessed. The selected patients had the following characteristics: Japanese-Brazilian descendent of parents and/or grandparents from Japan, except for the island of Okinawa, and Caucasian Brazilian parents (Portuguese, Spanish or Italian ancestry); aged between 11.91 to 16.61 years; with normal occlusion; and no history of previous orthodontic treatment.

All patients had permanent teeth in occlusion, except for third molars. Additionally, they had normal molar relationship, mild or absent crowding, no crossbite, normal overbite and overjet, no differences between mandibular positions in centric relation and maximum intercuspation, and well-balanced faces.

Cephalometric radiographs were obtained according to the standards recommended by the Department of Radiology, School of Dentistry/University of São Paulo, Bauru. Radiographic image magnification (using a Siemens equipment) was of 9.8%, corrected during measurements of radiographs so as to increase accuracy of the method employed. 

### Preparation of cephalograms

**Figure 1:**
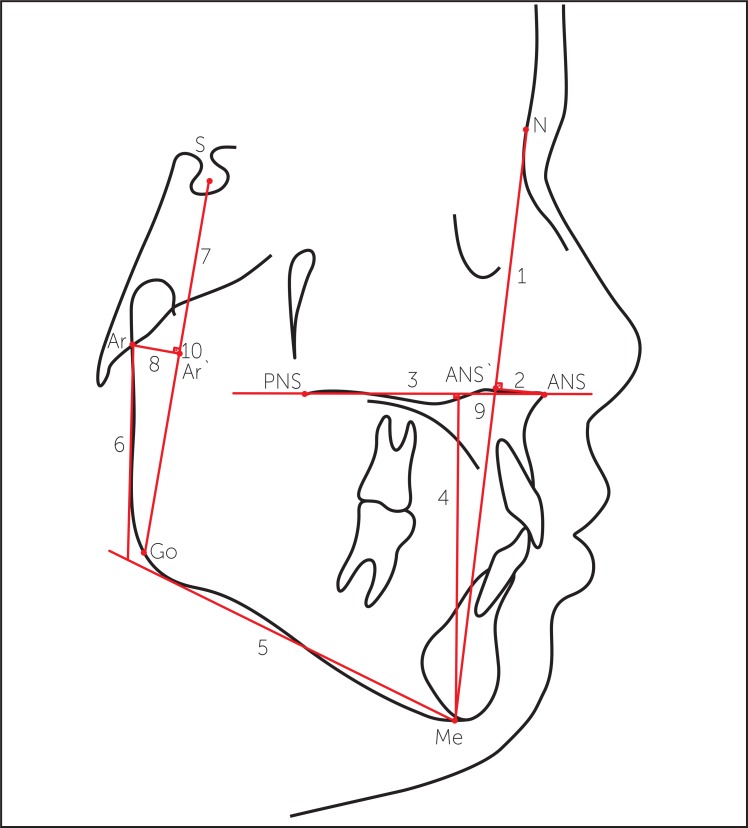
Cephalometric landmarks, lines and planes.

Anatomical tracing was carried out according to the recommendations described by Interlandi^11^ and Vion;^28^ the average of anatomical structures was used when two radiographic images of the same structure were identified. The following anatomical structures were assessed (Fig 1): Sella turcica, clivus, external cortex of the frontal bone and nasal bones; mean of pterygomaxillary fissure; mean of inferior borders of orbits; average of external auditory meatus; maxilla, mandible, teeth (upper and lower central incisors and first molars) and soft tissue profile.

After performing the anatomical tracing, landmarks were identified and then digitized by the digitizing tablet AccuGrid XNT, model A30TL.F (Numonics Corporation, Montgomeryville, PA, USA). Data were processed using Dentofacial Planner Software, version 7.02 (Dentofacial Planner Software Inc.,Toronto, Ontario, Canada) installed in a PC with 700MHz Intel Pentium III processor.

### Cephalometric landmarks, lines and planes

After the anatomical tracing was prepared, cephalometric landmarks were located according to Miyashita:^19^ S (Sella), N (Nasion), ANS (Anterior Nasal Spine), PNS (Posterior Nasal Spine), Me (Menton), Go (Gonion) and Ar (Articulare) (Fig 1). After locating the cephalometric landmarks that are independent of guidance tracing, plans and lines were drawn, and Ar' and ANS' points were constructed according to Wylie and Johnson^30^ as well as Siriwat and Jarabak^24^ (Fig 1).

1) N - Me line: The line formed by the union of Nasion (N) and Menton (Me).
2) ANS perp. line: The perpendicular line formed by the union of Anterior Nasal Spine (ANS) and N - Me line.
3) Palatal Plane (PP): The line formed by the union of Anterior Nasal Spine (ANS) and Posterior Nasal Spine (PNS).
4) Me - PP line: The line perpendicular to the Palatal Plane (PP) connecting this plan to the Menton (Me).
5) Mandibular Plane (MP): A line which bisects the distance between the left and right mandibular lower borders and connects anteriorly with Menton (Me).
6) Ar - MP line: The line connecting the Articulare (Ar) point to the Mandibular Plane (MP), touching the posterior border of the mandible branch.
7) S - Go line: The line formed by the union of Sella (S) and Gonion (Go).
8) Ar perp. line: The line formed by the projection of the Articulare (Ar) and perpendicular to S - Go line.
9) ANS' point (ANS projection point): Point formed by the intersection of ANS perp. line and N - Me line.
10) Ar' point (Ar projection point): the point formed by the intersection of Ar perp. line and S - Go line.

### Cephalometric measurements in vertical direction

**Figure 2:**
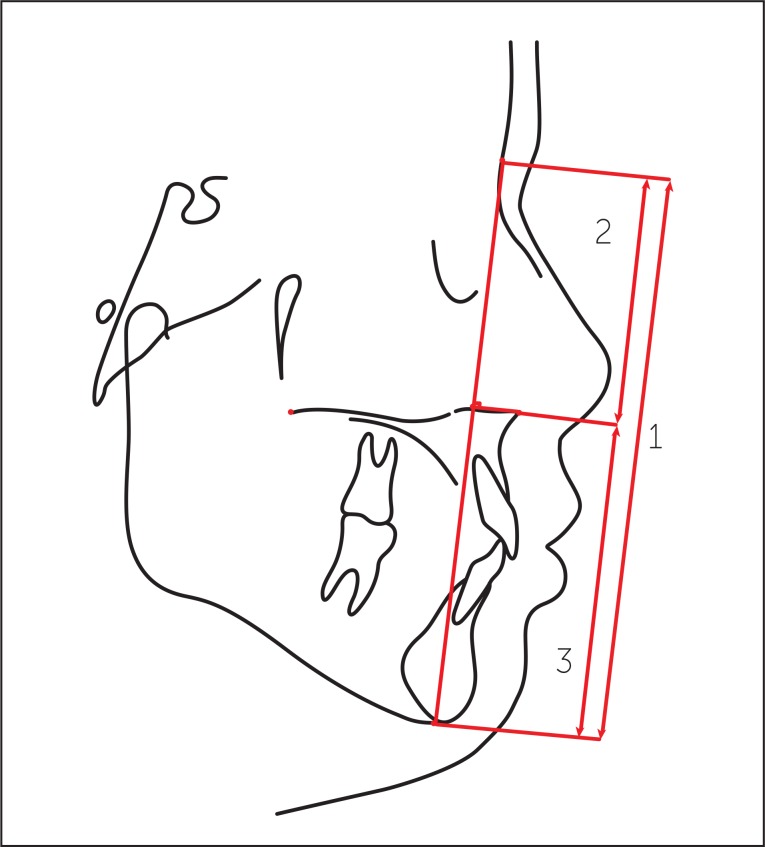
Measurements assessed, according to Wylie and Johnson analysis.

The measures taken according to the analysis by Wylie-Johnson^30^ are shown in Figure 2.

1) Total Anterior Facial Height (TAFH): Linear distance between Nasion (N) and Menton (Me).
2) Upper Anterior Facial Height (UAFH): Linear distance between points N and ANS', measured in N - Me line.
3) Lower Anterior Facial Height (LAFH): Linear distance between ANS' and Me, measured in N - Me line.
4) Ratio of Upper Anterior Facial Height and Total Anterior Facial Height (UAFH/TAFH).
5) Ratio of Lower Anterior Facial Height and Total Anterior Facial Height (LAFH/TAFH).

**Figure 3:**
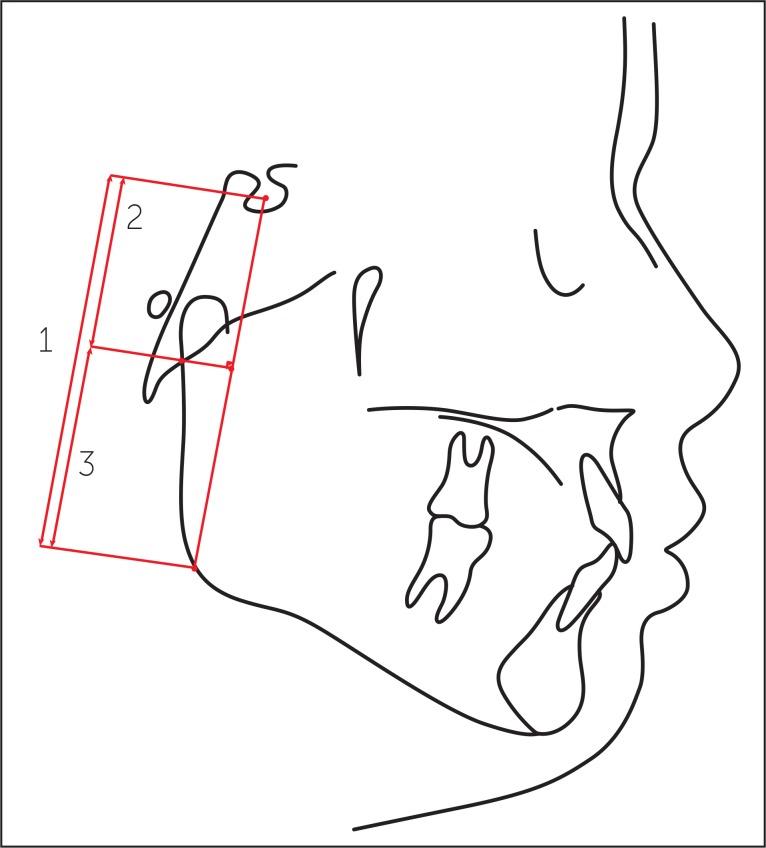
Measurements assessed, according to Siriwat and Jarabak analysis.24

The measures used according to the Siriwat and Jarabak^24^ analysis are shown in Figure 3.

1) Total Posterior Facial Height (TPFH): Linear distance between Sella (S) and Gonion (Go).
2) Upper Posterior Facial Height (UPFH): Linear distance between S and Ar' (perpendicular projection of Ar), measured in S-Go line.
3) Lower Posterior Facial Height (LPFH): Linear distance between Ar' and Go, measured in S-Go line.
4) UPFH/TPFH - Ratio of Upper Posterior Facial Height and Total Posterior Facial Height.
5) LPFH/TPFH - Ratio of Lower Posterior Facial Height and Total Posterior Facial Height.

**Figure 4:**
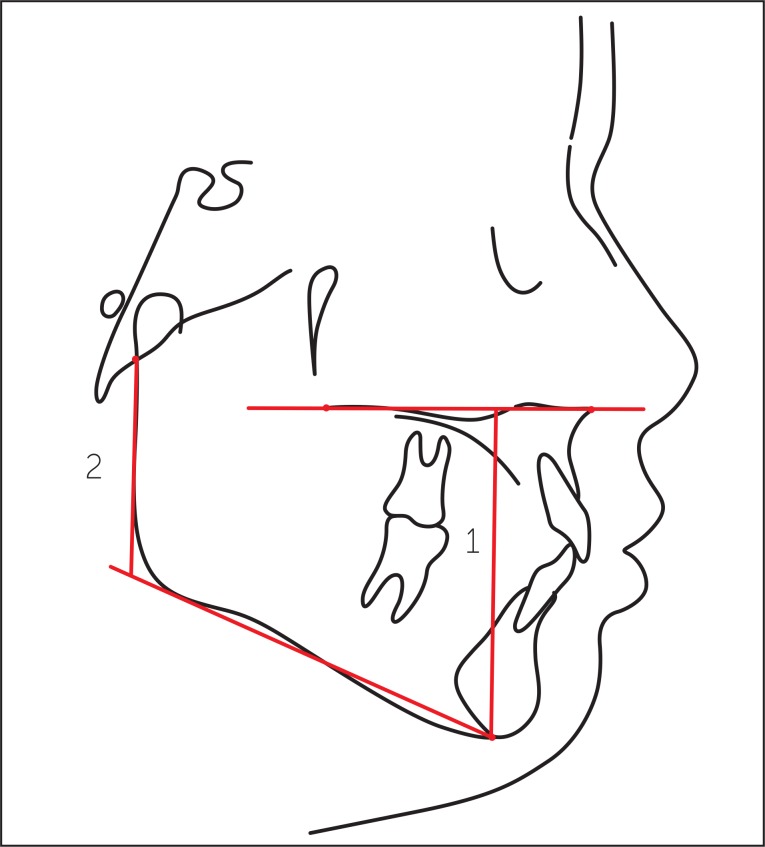
Measurements assessed, according to Gebeck, Merrifield and Horn analysis.8,9,17

The measures used according to the Gebeck^8 ^and Merrifield^17^ analysis and also used to determine the Facial Height Index of Horn^9^, are shown in Figure 4.

1) Anterior Facial Height (AFH): Perpendicular linear distance between Palatal Plane and Me, measured in Me - PP line.
2) Posterior Facial Height (PFH): Linear distance between Ar and the Mandibular Plane (Go-Me), tangent to the mandibular ramus.
3) Facial Height Index (FHI): Ratio of PFH and AFH, multiplied by 100 (FHI = PFH/AFH x 100)

## STATISTICAL METHOD

### Descriptive and comparative analyses

Means and standard deviation were used to describe the sample of Japanese-Brazilian descendants. To investigate the existence of sexual dimorphism, t-test with significance level set at 0.05 was applied due to normal distribution of variables in the Kolmogorov-Smirnov test. All statistical analyses were performed using Statistica software (Statistica for Windows 6.0, Statsoft, Tulsa, OK).

### Method error

Cephalometric tracings and measurements of 50% of the sample were remade by the same examiner a month after obtaining the initial cephalograms.

Systematic and casual errors were independently assessed for each cephalometric variable, as recommended by Houston.^10^ Systematic error was calculated by dependent t-test for paired samples. Casual error was calculated by Dahlberg's formula^4^ using the standard deviation of differences between repetitions.

## RESULTS

Results are divided and presented in tables for didactics purposes and to favor visualization and understanding.

## CASUAL AND SYSTEMATIC ERROR

Casual error was determined by Dahlberg's formula,^4^ whereas systematic error was assessed by dependent t-test.^10^ Statistical analysis carried out to assess intra-examiner error revealed no systematic errors. Casual errors, however, were minimal, since measurements were linear and most of them had a value lower than 1 mm. Only two variables, TAFH and UAFH, yielded slightly higher causal error values: 1.44 mm and 1.25 mm, respectively.

## CHARACTERIZATION OF THE SAMPLE

The sample comprised 30 Japanese-Brazilian descendants, 15 males and 15 females, with mean age of 14 years old - 14.78 years for males and 13.22 years for females, representing an age difference of 1.56 years of which significance was tested and confirmed by independent t-test set at 5% significance level. Characterization of vertical facial growth pattern by means of SN.GoGn variable showed an average of 33.08 degrees (33.02 for males and 33.15 for females), with no statistically significant difference. 

## SAMPLE COMPARATIVE AND DESCRIPTIVE ANALYSES

Descriptive analysis determined the number of research subjects, means, standard deviations as well as minimum and maximum values of the population necessary for a confidence interval of 95% for each variable considered in the current study (Table 1).

**Table 1:** Descriptive analysis of Japanese-Brazilian descendent sample.

Variable	n	Mean ± SD	Min-Max values for a Confidence Interval of 95%
TAFH	30	122.82 ± 7.54	120.00 – 125.63
UAFH	30	52.52 ± 4.08	50.99 – 54.04
LAFH	30	70.29 ± 4.81	68.49 – 72.09
UAFH/TAFH	30	42.76 ± 1.90	42.05 – 43.47
LAFH/TAFH	30	57.23 ± 1.90	56.52 – 57.94
TPFH	30	81.60 ± 5.47	79.56 – 83.64
UPFH	30	34.47 ± 3.58	33.13 – 35.81
LPFH	30	47.13 ± 3.89	45.67 – 48.58
UPFH/TPFH	30	42.23 ± 3.08	41.07 – 43.48
LPFH/TPFH	30	57.77 ± 3.08	56.61 – 58.92
AFH	30	69.45 ± 4.81	67.65 – 71.24
PFH	30	50.33 ± 4.03	48.83 – 51.84
FHI	30	72.65 ± 6.20	70.33 – 74.97

Sexual dimorphism was assessed by means of independent t-test set at 5% significance level. Average male and female data with respective standard deviations and P-values are presented in Table 2.

**Table 2:** Comparative analysis of male and female Japanese-Brazilian descendents.

Variable	Mean ± SD Female (n = 15)	Mean ± SD Male (n = 15)	P
TAFH	119.74 ± 4.20	125.90 ± 8.93	0.022*
UAFH	51.64 ± 3.66	53.40 ± 4.41	0.243
LAFH	68.10 ± 2.86	72.49 ± 5.42	0.009*
UAFH/TAFH	43.10 ± 2.15	42.42 ± 1.62	0.337
LAFH/TAFH	56.89 ± 2.15	57.57 ± 1.62	0.337
TPFH	78.85 ± 3.91	84.36 ± 5.51	0.003*
UPFH	32.70 ± 2.45	36.24 ± 3.71	0.004*
LPFH	46.14 ± 3.00	48.12 ± 4.50	0.168
UPFH/TPFH	41.48 ± 2.35	42.98 ± 3.60	0.190
LPFH/TPFH	58.51 ± 2.35	57.02 ± 3.61	0.192
AFH	67.38 ± 2.75	71.52 ± 5.58	0.015*
PFH	49.51 ± 3.29	51.16 ± 4.62	0.270
FHI	73.48 ± 4.28	71.82 ± 7.74	0.473

* Significant for P < 0.05.

## DISCUSSION

Facial vertical pattern affects facial harmony and attractiveness. In this context, orthodontic treatment can favor or disfavor balance by implementing facial changes in the vertical direction of which even lay people are aware of.^21^ Therefore, clinicians should have an individualized reference^20^ to conduct orthodontic treatment in order to induce the desired changes and minimize undesirable, inevitable ones.^2^

This study should be viewed as primarily descriptive. It aims at demonstrating how the values of young Japanese-Brazilian descendants are incomparable to values previously established for Caucasian and Mongoloid subjects. It also aims at analyzing sexual dimorphism for each variable. Thus, the values determined for the variables analyzed herein should be compared to other results previously reported in the literature with a view to further investigate this topic.

This discussion of results is divided into anterior facial height and its ratios, posterior facial height and its ratios, and Facial Height Index (Horn^9^). Each of these sections was divided into sub-sections so as to favor interpretation of results.

## ANTERIOR FACIAL HEIGHTS

### TAFH - Total anterior facial height

TAFH for Japanese-Brazilian descendants had an average value of 125.90 mm for males and 119.74 mm for females. Statistically significant difference, with significance level set at 5%, was identified between these values, thereby indicating sexual dimorphism with greater vertical development for males.

 This may have been caused by age difference between males and females. However, there is a chronological gap between growth and development of males and females in the phase of adolescence, including the vertical development of the face. Additionally, females in general have their pubertal growth spurt at an earlier age than males. Therefore, growth will likely be more balanced between males and females in this condition, with a mean age difference of 1.56 years during adolescence, particularly because females represented the group with the lower average age. Similar findings were also reported in other studies.^14^^,^ 26

The values determined for Japanese-Brazilian descendants are close to the highest values found in the literature for Caucasians, but were even closer to values found for Mongoloids. Ishii et al^12^ conducted a study in which significant differences were found between Japanese Mongoloid and British Caucasian groups for both males and females, with the Mongoloid group presenting the highest values. Takahashi^26^ also found significant differences between Caucasian and Mongoloid racial groups, particularly for males, with the largest values found in the Mongoloid group. However, for females, no significant differences were found among racial groups. Additionally, the female Mongoloid group had higher values of TAFH.

**Figure 5:**
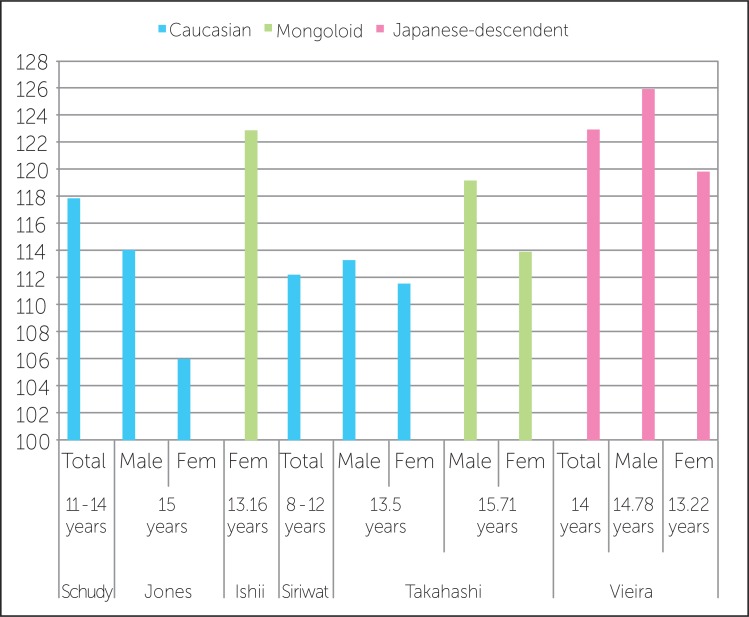
TAFH means.

Although the values found in the literature showed great variability for the TAFH variable, in general, the values reported in this study were very close to those found in the literature for Japanese^12^ and their descendents^26^ within a similar age range. Disagreement among some values found in the literature^12^^,^ 14 ^,^ 23 ^,^ 24 ^,^ 26 explains the large variation among them (Fig 5).

### UAFH - Upper anterior facial height

Young Japanese-Brazilian descendants showed an average UAFH value of 52.52 mm (53.40 mm for males and 51.64 mm for females) with no statistically significant difference at 0.05 significance level between them. Thus, sexual dimorphism was not evident, thereby implying that upper facial height does not contribute to dimorphism found in TAFH.

Results showed no differences between males and females, confirming the findings by Domiti et al^5^ and Locks.^15^ However, other authors, such as Jones and Meredith^14^ as well as Ursi et al^27^ found a higher value for the upper anterior facial height for males. Additionally, Takahashi^26^ found a higher value for the Mongoloid group, but not for the Caucasian one.

The values for young Japanese-Brazilian descendants are between those found in the literature for Caucasians,^6^^,^ 27 but once more are closer to those reported by Takahashi^26^ for the Mongoloid group.

Ishii et al^13^ showed that the upper anterior facial height was significantly higher in the Japanese Mongoloid group in comparison to the British Caucasian group. Takahashi^26^ found a significant difference comparing Caucasian and Mongoloid racial groups for males, but not for females. He also observed higher values for the Mongoloid group when comparing males and females of both races.

**Figure 6:**
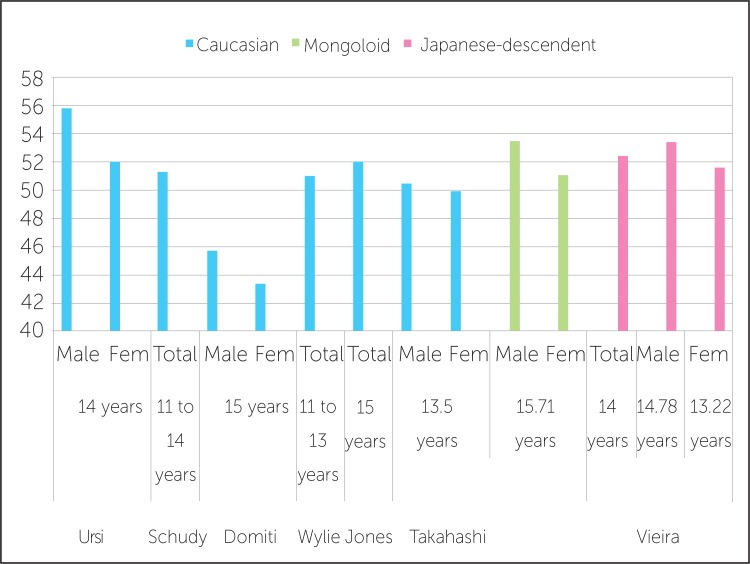
Means of UAFH.

Figure 6 shows the values found in the literature for UAFH.^5^^,^ 14 ^,^ 23 ^,^ 26 ^,^ 27 ^,^ 29

### LAFH - Lower anterior facial height

The mean LAFH values for young Japanese-Brazilian descendants are 70.29 mm, 72.49 mm for males and 68.1 mm for females. Values were statistically different for males and females, thereby featuring sexual dimorphism and confirming the findings by other authors such as Lock^15^ and Miyajima.^18^ However, Domiti et al^5^ and Takahashi^26^ found no differences between males and females for either one of the two racial groups. Ursi et al^27^ identified differences between males and females older than 16 years with LAFH values higher for males at this age. Sexual dimorphism in the Japanese-Brazilian descendent sample leads us to the conclusion that lower anterior facial height contributed significantly to the dimorphism found in TAFH.

The values determined for the young Japanese-Brazilian descendants are closely related to the highest values found in the literature for Caucasians^6^ and Mongoloids.^18^

Ishii et al^13^ reported that Japanese Mongoloid individuals have LAFH values significantly higher than British Caucasian individuals, although Takahashi^26^ found significant differences between Caucasian and Mongoloid racial groups, only for males, thereby demonstrating greater LAFH values in the Mongoloid group for both males and females.

**Figure 7:**
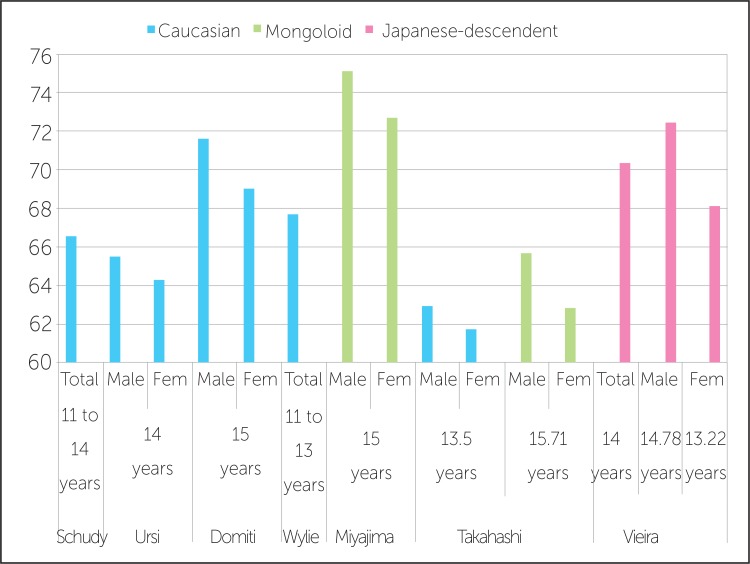
Means of LAFH.

Figure 7 shows the values found in the literature^5^^,^ 18 ^,^ 23 ^,^ 26 ^,^ 27 ^,^ 29 with a large variation for LAFH values.

### UAFH/TAFH ratio

Young Japanese-Brazilian descendants showed an average UAFH/TAFH ratio of 42.76%, being 43.10% for females and 42.42% for males, with no statistically significant difference between these values, at 0.05 significance level. Consequently, no sexual dimorphism was evident, corroborating the findings by Wylie and Johnson,^30^ as well as Takahashi^26^ - who did not find a statistically significant difference between males and females for both study groups at 0.05 significance level.

The values determined for young Japanese-Brazilian descendants are closer to the minimum value found by Locks^16^ for Caucasians (42%).

Takahashi^26^ also found no significant differences when comparing Caucasian and Mongoloid males and females, thus showing a balance in this ratio.

**Figure 8:**
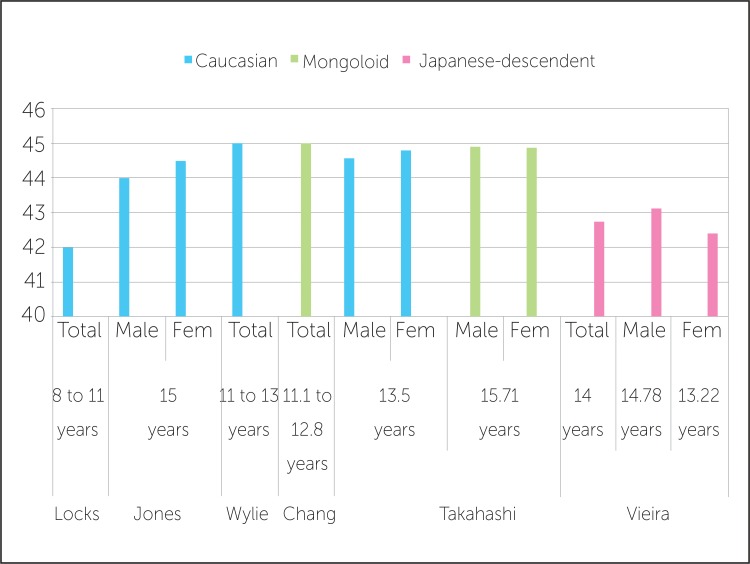
Means of UAFH - TAFH.

Figure 8 shows the comparison among values found in the literature.^3^^,^ 14 ^,^ 16 ^,^ 26 ^,^ 30

### LAFH/TAFH ratio

Young Japanese-Brazilian descendents showed an average UAFH/TAFH ratio of 57.23%, being 56.89% for females and 57.57% for males, with no statistically significant difference between these values, at a 0.05 significance level. Consequently, no sexual dimorphism was evident, corroborating the findings by Takahashi^26^ who found no statistically significant difference between males and females for both study groups.

The values determined for young Japanese-Brazilian descendants are close to the highest values found in the literature for Caucasians.^16^^,^ 23

Takahashi^26^ also found no significant differences when comparing Caucasian and Mongoloid males and females, thus showing a balance in this ratio.

**Figure 9:**
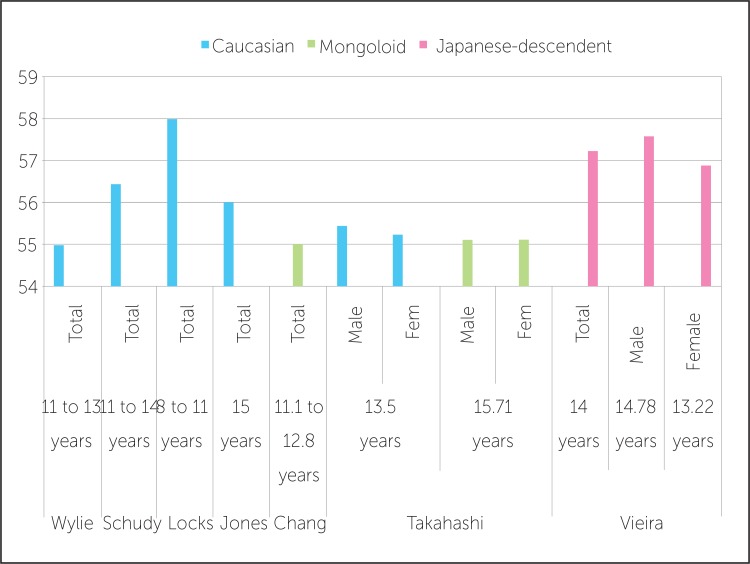
Means of LAFH - TAFH.

Figure 9 shows the values found in the literature for LAFH/TAFH.^3^^,^ 14 ^,^ 16 ^,^ 23 ^,^ 26 ^,^ 30

## POSTERIOR FACIAL HEIGHTS

### TPFH - Total posterior facial height

Young Japanese-Brazilian descendants showed an average TPFH of 81.60 mm, being 84.36 mm for males and 78.85 mm for females, with a statistically significant difference at a 0.05 level, thereby indicating sexual dimorphism with greater development of male posterior facial height. As discussed regarding TAFH dimorphism, it can be inferred that this is not a simple reflection of age difference between males and females. In addition, a similar condition was reported by Takahashi^26^ for the Mongoloid group, although Chang et al^3^ did not find this difference.

The values determined for young Japanese-Brazilian descendants are closer to the values found by Takahashi^26^ for the Mongoloid group. This author also found significant differences when comparing Caucasian and Mongoloid males and females, and reported that the Mongoloid group had higher values^26^. 

**Figure 10:**
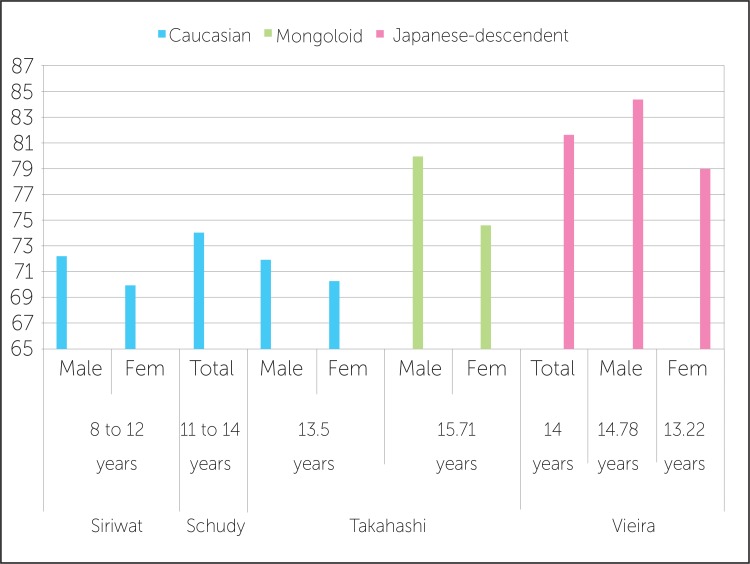
Means of TPFH.

The values reported in the literature for TPFH^23^^,^ 24 ^,^ 26 are shown in Figure 10.

### UPFH - Upper posterior facial height

The mean UPFH value of young Japanese-Brazilian descendants was 34.47 mm, being 36.24 mm for males and 32.70 mm for females. Values were statistically different for males and females with significance level set at 0.05. Thus, sexual dimorphism was characterized with high values for male posterior facial height. The upper portion of the posterior facial height can be inferred to contribute significantly to the dimorphism found in TPFH. Takahashi^26^ also reported the presence of sexual dimorphism for both Caucasian and Mongoloid groups, in addition to a greater vertical development of male upper posterior facial height for both groups.

Takahashi^26^ also identified significant differences when comparing Caucasian and Mongoloid racial groups, for males and females, with higher values for Mongoloids. These findings differed from those by Ishii et al^13^ who found no difference between Japanese Mongoloid and British Caucasian.

**Figure 11:**
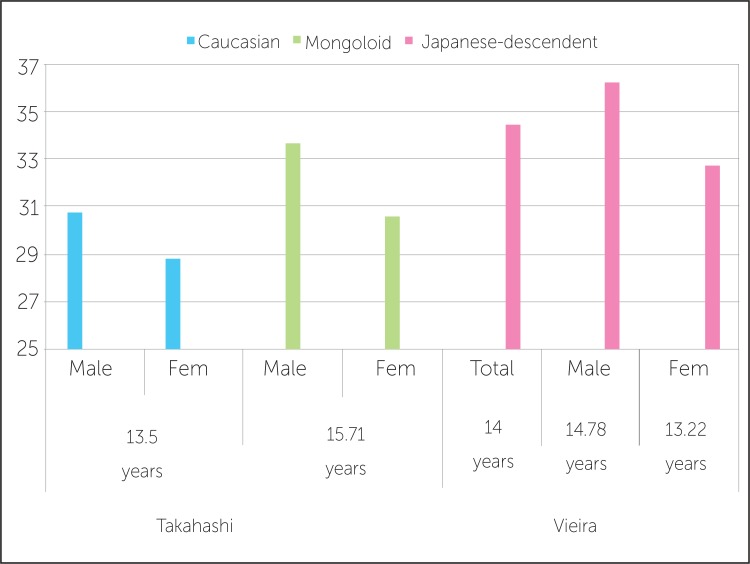
Means of UPFH.

Comparison between values found in this study and by Takahashi^26^ indicate greater proximity between the values of young Japanese-Brazilian descendants and Mongoloids, with higher values for the first group, as presented in Figure 11. This finding can neither be attributed to differences in methodology, which wa s the same, nor to mean age difference, since the mean age of the Mongoloid group was greater (15.71 years) in Takahashi's study^26^. However, this finding may be due to the use of a different sample, with a slightly more vertical pattern of young Japanese-Brazilian descendants, or because of race miscegenation that generates a new biological and genetic conformation.

### LPFH - Lower posterior facial height

Young Japanese-Brazilian descendants showed an average LPFH value of 47.13 mm, being 48.12 mm for males and 46.14 mm for females. No sexual dimorphism was evident, thereby corroborating the results by Takahashi^26^ f r both groups.

Lack of dimorphism in LPFH values of young Japanese-Brazilian descendants inferred that LPFH does not contribute to the dimorphism found in TPFH.

**Figure 12:**
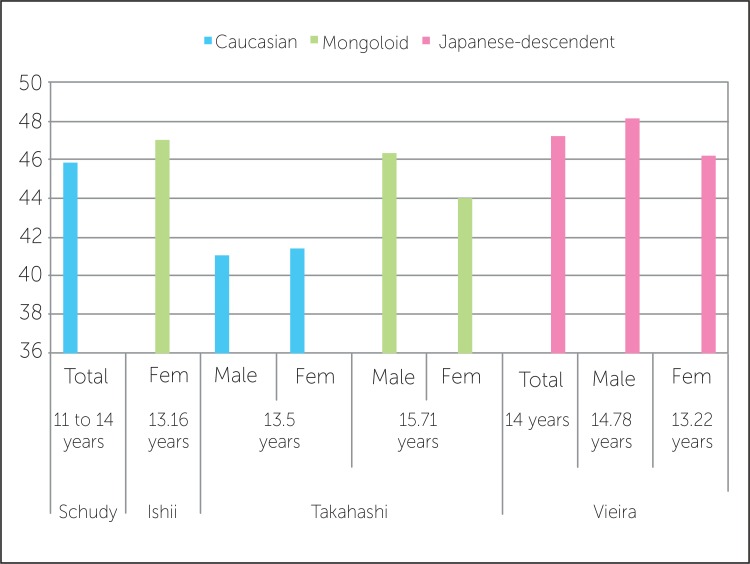
Means of LPFH.

Takahashi^26^ identified significant differences when comparing Mongoloid and Caucasian males and females, with the Mongoloid group showing higher values. The values of young Japanese-Brazilian descendants are closer to the maximum values obtained with Caucasians,^23^ and even closer to the values reported for Mongoloids.^12^^,^ 26 This condition is well characterized in Figure 12.

### UPFH/TPFH ratio

Young Japanese-Brazilian descendants showed an average UPFH/TPFH ratio of 42.23%, being 42.98% for males and 41.48% for females, with no statistically significant difference at a 0.05 significance level. Therefore, no sexual dimorphism was observed. This result corroborates the findings by Takahashi^26^ for the Mongoloid group, although this author reported sexual dimorphism with higher UPFH/TPFH ratios for males in the Caucasian group.

**Figure 13:**
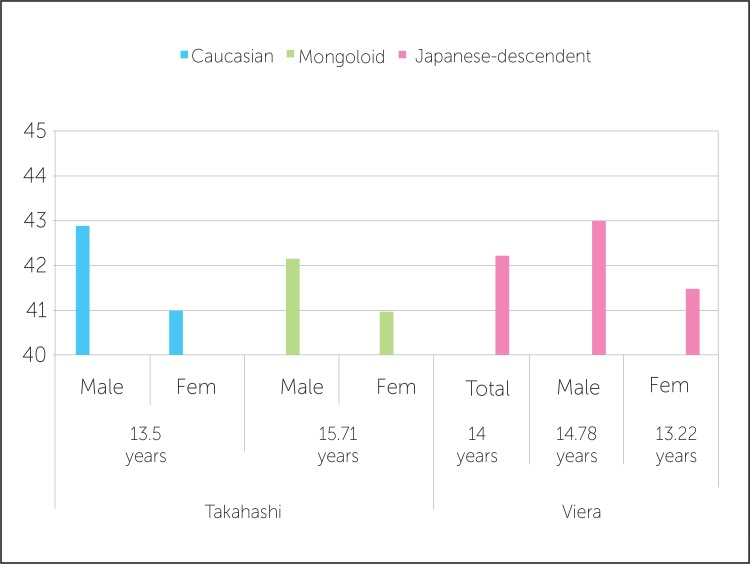
Means of UPFH - TPFH.

Takahashi^26^ found no significant difference when comparing Caucasian and Mongoloid males and females. The values for young Japanese-Brazilian descendants are close to those reported by Takahashi,^26^ as shown in Figure 13, despite age difference and the use of a different sample with its own racial miscegenation. The cause may be stability of values for this variable after a certain age and a small variation between different races and their miscegenations.

### LPFH/TPFH ratio

Young Japanese-Brazilian descendants showed an average LPFH/TPFH value of 57.77%, being 57.02% for males and 58.51% for females. The lack of dimorphism in these results corroborates Takahashi^26^ for the Mongoloid group, although this author reported sexual dimorphism in the Caucasian group with higher LPFH/TPFH values for females.

**Figure 14:**
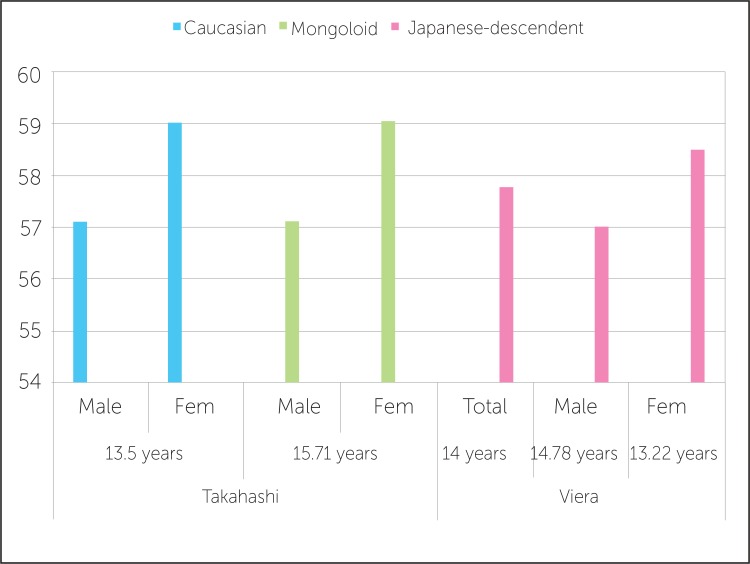
Means of LPFH - TPFH.

Takahashi^26^ found no significant differences when comparing Mongoloid and Caucasian males and females. The values for young Japanese-Brazilian descendants are close to those reported by Takahashi^26^ for the two groups, as shown in Figure 14.

### Determining the facial height index (FHI)

Horn^9^ proposed a variable to track patient's vertical dimension during treatment. The proposed index is calculated by dividing the posterior facial height (PFH, the distance in millimeters from point Ar to the mandibular plane) and the anterior facial height (AFH) (the distance in millimeters from the palatal plane to the point Me). According to the author, the use of the facial height index^9^ is an additional aid in the diagnosis of excess or reduced vertical dimension, allowing observation of vertical dimension during treatment and adjustment of orthodontic mechanics to offset any unfavorable trend.

### AFH - Anterior facial height

The mean AFH value of young Japanese-Brazilian descendants was 69.45 mm, being 71.52 mm for males and 67.38 mm for females. Sexual dimorphism was identified, with larger vertical development of the male group. This result appears to be consistent with LAFH and TAFH values and also corroborates the findings by Takahashi^26^ in the Mongoloid group, with more vertical development of the anterior facial height of males, although the same author reported the absence of dimorphism in the Caucasian group.

**Figure 15:**
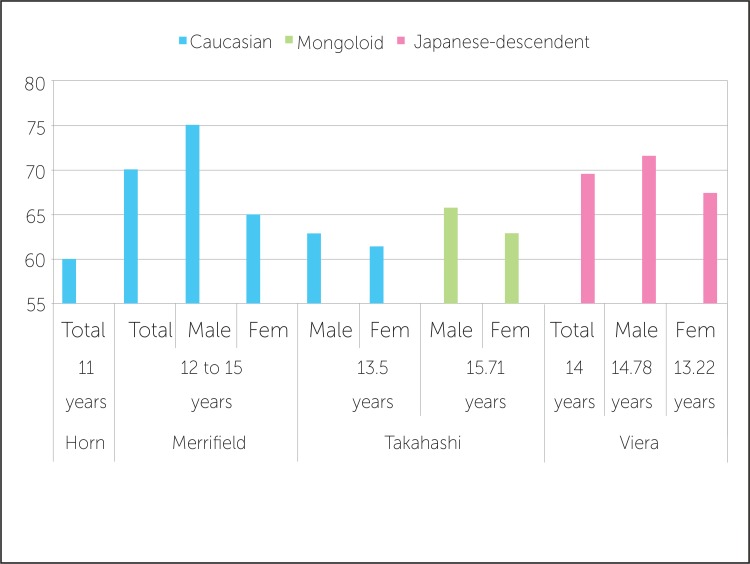
Means of AFH.

The values determined for young Japanese-Brazilian descendants are closer to the maximum values found in the literature for Caucasians,^9^^,^ 17 and closer to the values reported by Takahashi^26^ for the Mongoloid group. The variation in AFH found in the literature is shown in Figure 15.

Takahashi^26^ identified significant differences when comparing Caucasian and Mongoloid males, and found no significant differences among racial groups for females. The same author also reported that, with regard to both males and females, Mongoloids had higher values than Caucasians.

### PFH - Posterior facial height

The mean PFH value of young Japanese-Brazilian descendants was 50.33 mm, being 51.16 mm for males and 49.51 mm for females. No sexual dimorphism was found with these results, similar to what was observed in Takahashi's^26^ study for both Mongoloid and Caucasian groups.

**Figure 16:**
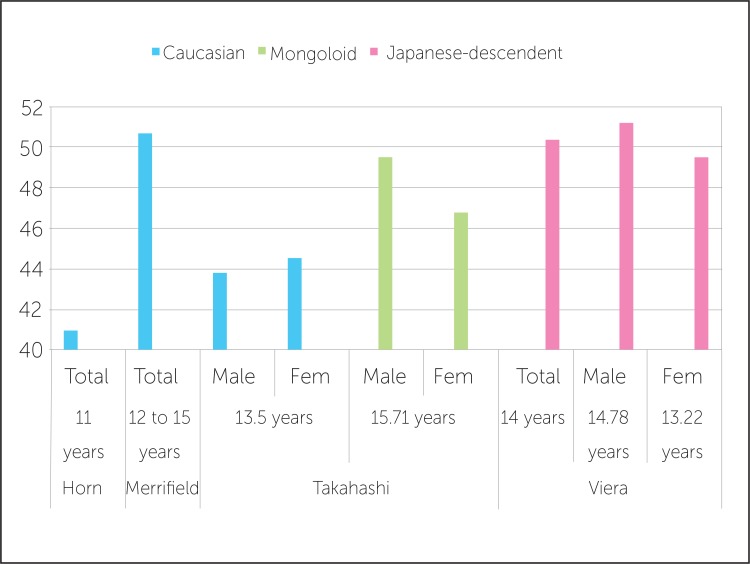
Means of PFH.

The values determined for young Japanese-Brazilian descendants are close to those found in the literature for Caucasians^17^ and even closer to those reported for the Mongoloid group.^26^ Takahashi^26^ also reported significant difference when comparing Caucasian and Mongoloid males and females. The variation in PFH values found in the literature^9^^,^ 17 is shown in Figure 16.

### FHI - Facial Height Index (Horn^9^)

The mean FHI value for Japanese-Brazilian descendants (Horn^9^) was 72.65%, being 71.82% for males and 73.48% for females. No sexual dimorphism was found, thereby corroborating the results by Takahashi^26^ for the Mongoloid group, although this author reported sexual dimorphism in the Caucasian group.

**Figure 17:**
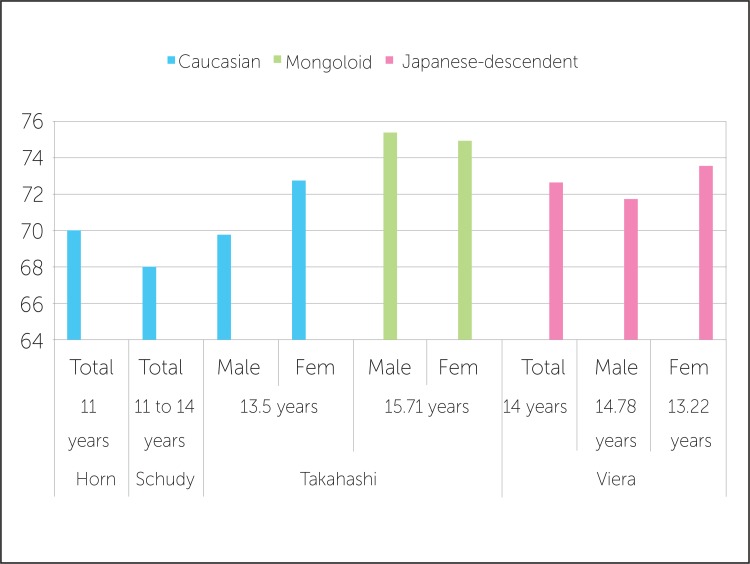
Means of FHI.

A small variation in FHI values was observed in the literature,^9^^,^ 23 ^,^ 26 as shown in Figure 17. For this variable, the results of the present study were similar to those reported by Takahashi^26^ for Caucasians and Mongoloids. He also reported significant differences between Caucasian males, but not for females.

Results for young Japanese-Brazilian descendants showed, in general, that the values of variables and the analysis of sexual dimorphism - particularly when compared with those reported by Takahashi^26^ - suggested a closer relationship between the values of Japanese-Brazilian descendants and the Mongoloid group for all variables except for the ratios UAFH / TAFH, LAFH / TAFH, UPFH / TPFH, and LPFH / TPFH as well as FHI. Thus, further comparison between young Japanese-Brazilian descendants, Caucasians and Mongoloids should be performed in a study with the specific objective of precisely establishing the relationship between these groups.

Moreover, the analysis of sexual dimorphism of young Japanese-Brazilian descendants suggests a more vertical pattern for males, characterized by higher values in the variables TAFH, LAFH, AFH, TPFH and UPFH. However, the variables denoting vertical facial ratio do not show the same trend.

## CLINICAL CONSIDERATIONS

Vertical facial changes influence mandibular position and rotation, either clockwise or counterclockwise,^22^^,^ 23 thereby contributing to the development of deep bite or open bite, and potentially increasing the severity of anterior-posterior malocclusion. Thus, orthodontic treatment should induce desirable alterations and minimize the undesirable ones when the latter are inevitable.^2^

Therefore, malocclusion should be analyzed completely and in all different dimensions of space so as to favor understanding of how and in what direction each element of the stomatognathic system contributes to the conformation of malocclusion, which is necessary for cephalometric analysis of anterior-posterior and vertical directions, as well as analysis of the influence of vertical changes in the severity of antero-posterior malocclusion.^23^

However, the literature indicates that malocclusion analysis requires full assessment so as to individualize cephalometric norms regarding patient's sex, age and race.^20^ Thus, cephalometric standards from different ethnic and racial groups and miscegenations were determined and compared, and the need for individualization for each specific group was demonstrated^5^^,^ 7 ^,^ 18 ^,^ 20 ^,^ 26 to better understand and assess the cephalometric characteristics of different groups and miscegenations with respect to orthodontic diagnosis and planning. Thus, the present study provides the clinician with a more specific reference in the vertical direction of the face, particularly for young Japanese-Brazilian descendants with normal occlusion.

Moreover, as a topic for future research, the values of Japanese-Brazilian descendants should be compared with those of other subjects, particularly Mongoloid and Caucasian Brazilians.

## CONCLUSIONS

Based on the sample and methods employed herein, values are presented to establish a cephalometric pattern of anterior and posterior facial heights and its ratios, as well as the facial height index (Horn^9^) for young Japanese-Brazilian descendants with normal occlusion. Results revealed the presence of sexual dimorphism in the following cephalometric measurements: TAFH, LAFH, AFH, TPFH and UPFH.
